# Heterocyclic Substitutions Greatly Improve Affinity and Stability of Folic Acid towards FRα. an In Silico Insight

**DOI:** 10.3390/molecules26041079

**Published:** 2021-02-18

**Authors:** Mohammad G. Al-Thiabat, Fadi G. Saqallah, Amirah Mohd Gazzali, Noratiqah Mohtar, Beow Keat Yap, Yee Siew Choong, Habibah A Wahab

**Affiliations:** 1 School of Pharmaceutical Sciences, Universiti Sains Malaysia, Gelugor 11800, Penang, Malaysia; mohd.althiabat@gmail.com (M.G.A.-T.); fadi_saqallah@student.usm.my (F.G.S.); amirahmg@usm.my (A.M.G.); noratiqah@usm.my (N.M.); Beowkeat@usm.my (B.K.Y.); 2 Pharmaceutical Design and Simulation (PhDS) Laboratory, Universiti Sains Malaysia, Gelugor 11800, Penang, Malaysia; 3 Institute for Research in Molecular Medicine (INFORMM), Universiti Sains Malaysia, Gelugor 11800, Penang, Malaysia

**Keywords:** folate receptor alpha, folic acid and antifolates, molecular docking, molecular dynamics, MM-PBSA, H-bonds, POVME calculations, ADMET prediction

## Abstract

Folate receptor alpha (FRα) is known as a biological marker for many cancers due to its overexpression in cancerous epithelial tissue. The folic acid (FA) binding affinity to the FRα active site provides a basis for designing more specific targets for FRα. Heterocyclic rings have been shown to interact with many receptors and are important to the metabolism and biological processes within the body. Nineteen FA analogs with substitution with various heterocyclic rings were designed to have higher affinity toward FRα. Molecular docking was used to study the binding affinity of designed analogs compared to FA, methotrexate (MTX), and pemetrexed (PTX). Out of 19 FA analogs, analogs with a tetrazole ring (FOL03) and benzothiophene ring (FOL08) showed the most negative binding energy and were able to interact with ASP81 and SER174 through hydrogen bonds and hydrophobic interactions with amino acids of the active site. Hence, 100 ns molecular dynamics (MD) simulations were carried out for FOL03, FOL08 compared to FA, MTX, and PTX. The root mean square deviation (RMSD) and root mean square fluctuation (RMSF) of FOL03 and FOL08 showed an apparent convergence similar to that of FA, and both of them entered the binding pocket (active site) from the pteridine part, while the glutamic part was stuck at the FRα pocket entrance during the MD simulations. Molecular mechanics Poisson-Boltzmann surface accessible (MM-PBSA) and H-bond analysis revealed that FOL03 and FOL08 created more negative free binding and electrostatic energy compared to FA and PTX, and both formed stronger H-bond interactions with ASP81 than FA with excellent H-bond profiles that led them to become bound tightly in the pocket. In addition, pocket volume calculations showed that the volumes of active site for FOL03 and FOL08 inside the FRα pocket were smaller than the FA–FRα system, indicating strong interactions between the protein active site residues with these new FA analogs compared to FA during the MD simulations.

## 1. Introduction

Cancer is one of the most dangerous and prevalent diseases that attacks any part or organ in the body. It is characterized by irregular and uncontrollable growth of cells further than their typical limits. This unrestrained growth can spread and further expand to other organs [1]. Folate receptor (FR) is a type of receptor known for its abundant availability in epithelial malignancy cells [2,3,4]. It is a membrane-bound protein that binds to folate with high affinity at a low physiological concentration (Kd: <1 nM) [3,5,6,7]. There are four human FR isoforms (FRα, FRβ, FRγ, and FRδ) [6,8]. The FRα isoform is the most common isoform on the cancer cell surface [8,9] and widely expressed in cancers of epithelial tissues, including lung, breast, kidney, and ovarian cancers [10,11,12,13]. The extensive FRα expression during advanced stages of numerous cancers is needed to meet the folate requirements of the rapid cell division under the effect of low-folate concentration conditions [14]. Thus, many studies have looked into the potential of overexpressed FR as an interesting target, allowing its exploitation in cancer diagnostics [15,16] and targeted nano-drug delivery [17,18].

FA can be actively transported into cells by the reduced folate carrier (RFC) or via the folate receptors (FRs) either by photocytosis or endocytosis [19,20]. The affinity of FA to bind FRα is 100–200 times greater than to RFC [21]. The mechanism by which FA is incorporated into the folate receptor (Figure S1, Supplementary Materials) would be valuable knowledge in understanding the binding process and can be used for the binding of competitive drugs [22,23]. The folate receptor is a globular-like protein stabilized by disulfide bonds. It has four long α-helices (α-1, α-2, α-3, and α-6), two short α-helices (α-4, α-5), four short β-strands (β1-β4), and several loop areas [22]. The FRα binding pocket consists of a large number of tryptophan residues that can create a large hydrophobic environment to fit the aromatic folate component [22]. In addition, it has also several cysteine residues which can bind with high affinity with FA to facilitate its cellular uptake [24]. Several studies have shifted focus to the FRα isoform as a molecular target in many cancers, including studies on FRα antibodies, high-affinity antifolates, folate-based imaging agents, folate-conjugated drugs, and folate-conjugated nanoparticle delivery systems [22,25,26,27].

Antifolates are a group of drugs that block the action of FA inside the cell by inhibiting several enzymes such as dihydrofolate reductase (DHFR) and/or thymidylate synthase (TS) [28,29,30]. In recent clinical studies, antifolates such as methotrexate (MTX), pemetrexed (PTX), pralatrexate (PDX), raltitrexed (RTX), and edatrexate (EDX) bound to FRα and killed cancer cells [30,31,32]. These studies also showed that some of the antifolates, such as PTX, have better binding affinity towards FRα than FA [30,31,32], while some, such as MTX, RTX, and PDX, have comparable binding affinity to FA to the receptor [6,33]. It is highly possible that the functional groups present in each antifolate play an important role in the binding of the antifolate molecules with the receptor. However, the stereochemistry of the structures, the distance of each interaction, and the amino acids inside the receptor pocket also have a role in ligand–receptor binding [34].

Between 2010 and 2015, two-thirds of FDA-approved anticancer drugs contained heterocyclic rings in their chemical structure [35]. Heterocyclic rings have been shown to interact with many receptors and are important to the metabolism and biological processes within the body [36,37]. They differ in the ring size and heteroatomic structure, making them distinct in their interactions from weak interactions (such as hydrophobic, pi stack, and van der Waals) to strong interactions (such as ionic and H-bonds) [35,38]. The scientific literature over the last 10 years has shown that all new DHFR inhibitors of antifolate drugs are characterized by heterocyclic rings in their structure, which play a key role in increasing the affinity towards the folate receptor as well as in the inhibition of enzymes in cancer cells [29,39,40,41]. Multi-target drug in this scenario is a promising approach for new antifolate drugs.

**Figure 1:**
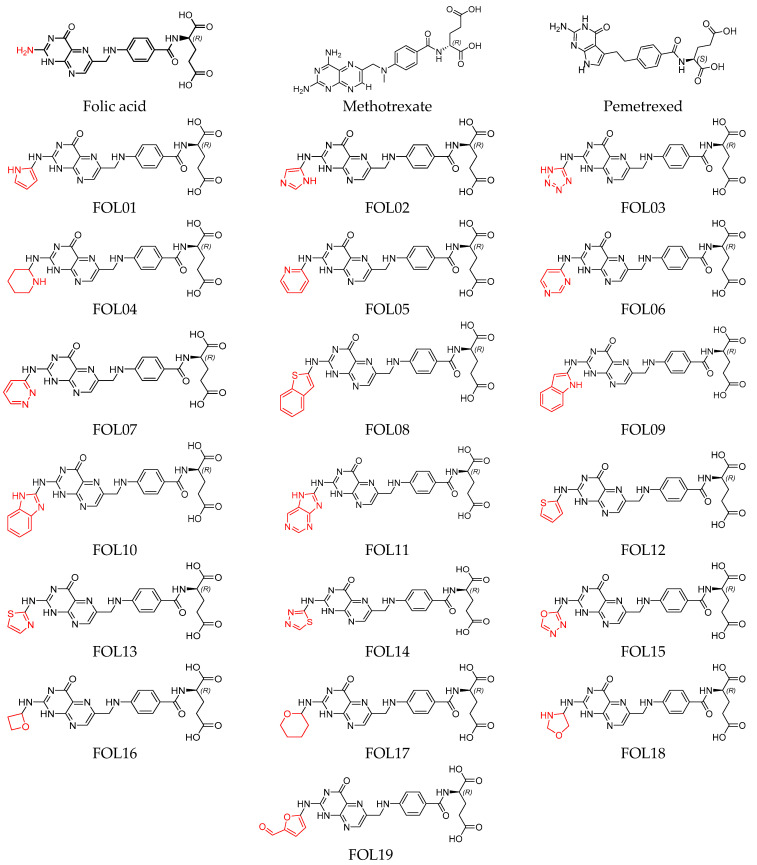
Illustration of the structures for folic acid (FA), methotrexate (MTX), pemetrexed (PTX) and the newly designed FA analogs (FOL1–19). The chemical structure in red is the heterocyclic ring attached to FA.

In the present study, nineteen new FA analogs were designed containing heterocyclic rings recently incorporated in anticancer agents and their effect on the binding affinity towards FRα was investigated (Figure 1). These analogs were compared with FA, MTX, and PTX. Analogs with the best affinity for interaction with FRα were selected for a molecular dynamics study, where more details on the binding mechanism were explored.

## 2. Results and Discussion

### 2.1. Investigation of FRα Binding Site

**Figure 2:**
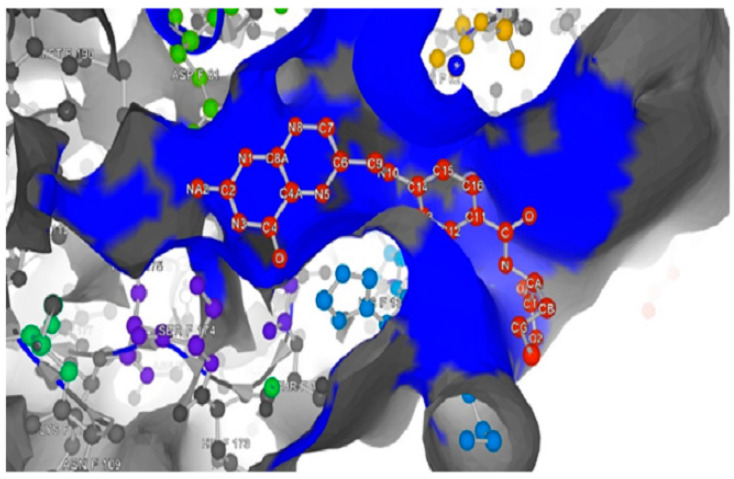
P2Rank prediction for folate receptor α (FRα) complex with FA (PDB:4LRH), the prediction of the binding pocket is indicated by a blue color and the FA is shown with red balls and gray sticks.

**Figure 3:**
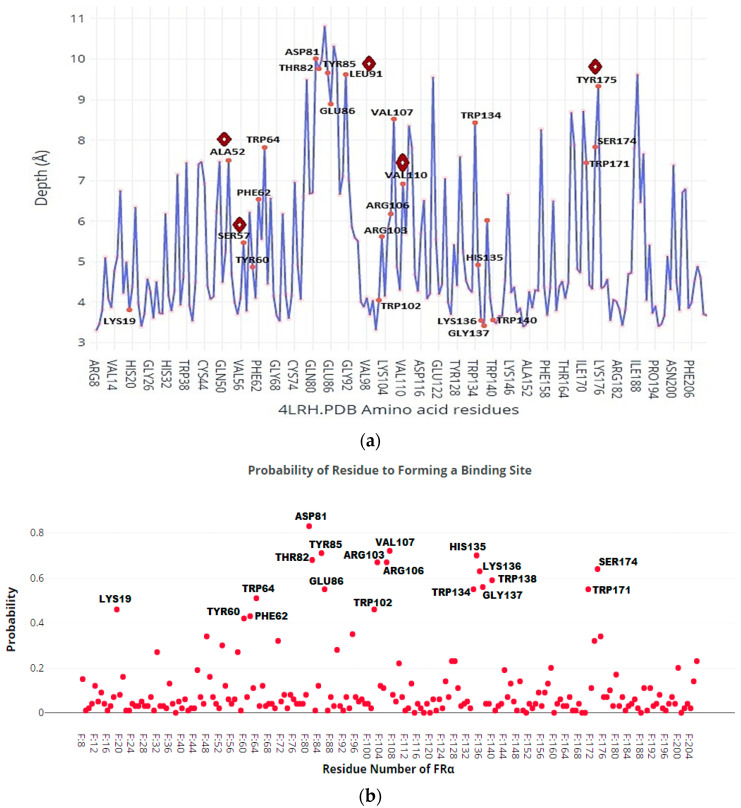
The amino acids existing in the depth of the pocket using Depth test. (**a**) 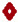 Unpredictable residues by depth server and confirmed by P2Rank server to form FRα active site. (**b**) The probability of amino acids, which exist in the binding pocket, forming the binding site.

The P2Rank online service performs rapid ligand-binding site prediction and gives visual results of the structure sequence [40,42]. Likewise, the Depth server measures the binding cavity volumes and predicted the location of binding sites by measuring the closest distance between an amino acid residue/atom to the bulk water molecule [43]. Figure 2 and Figure 3 demonstrate, respectively, the P2Rank binding site prediction for all the amino acid residues in the protein and Depth’s probability of the amino acid residues forming an FRα cavity. According to the P2Rank results, the active site of FRα is made up of ALA52, ASP81, GLU86, ARG103, ARG106, VAL107, VAL110, HIS135, LYS136, GLY137, TRP64, TRP102, TRP134, TRP138, TRP140, TRP171, SER57, SER174, TYR60, TYR85, TYR175, LYS19, PHE62, THR82, and LEU91. Twenty of the twenty-five amino acids predicted were supported by many previous studies [6,22,44]. The other five unreported amino acids (VAL107, VAL110, LEU91, LYS19, and THR82) could also lead to the formation of the active site, as demonstrated by P2Rank.

Figure 3 shows the residues in the binding site and their depth inside the pocket, as well as the probability of these amino acids participating in the creation of the FRα active site computed by Depth. The findings showed that the depth of the binding pocket extended to 11 Å (Figure 3a). Furthermore, the analysis shows that ASP81 is located deepest (~10 Å) within the FRα pocket and has the highest probability to be part of the active site (Figure 3b). In addition, other amino acids near ASP81, i.e., THR82, TYR85, VAL107, and SER174, are also found at pocket depths of 8.5–10 Å with a high likelihood of forming the active site compared to other amino acids in the site. Analysis of the binding pocket by P2Rank and Depth servers interestingly showed that there exists a small extra space in the depth of the pocket created by polar amino acids ASP81, THR82, TYR85, SER174, and non-polar amino acid VAL107. However, the presence of LEU91 and TYR175 in the depth of the pocket as P2Rank predicted was not confirmed by Depth. Nonetheless, the docking of FA confirmed that LEU91 is located in the binding site but is unable to establish interactions with the ligand, unlike ASP81 and TYR85, which could establish interactions with FA [22].

It is observed from Figure 2 and Figure 3 that there is a large space untapped at the depth of FRα’s active site. The extra small space provides an opportunity to modify the pteridine site of FA with different heterocyclic rings in order to improve the interactions with the amino acids in the pocket cavity. The findings of the earlier studies with the P2Rank and Depth servers did not vary substantially in terms of the reported amino acids [6,22,44]. However, those studies failed to identify LEU91, VAL107, and TYR175 which neighbor ASP81 at the depth of 8.5–10 Å.

### 2.2. Molecular Docking

Redocking FA into the co-crystallized FRα structure gave an RMSD value of 0.90 Å. RMSD value ≤ 1.0 Å is generally considered acceptable [45], thus the docking parameters used in the redocking of FA were extended to the docking of other ligands. The comparison between the docking conformations of the co-crystallized structures and the original docked FA is presented in Figure S2.

**Figure 4:**
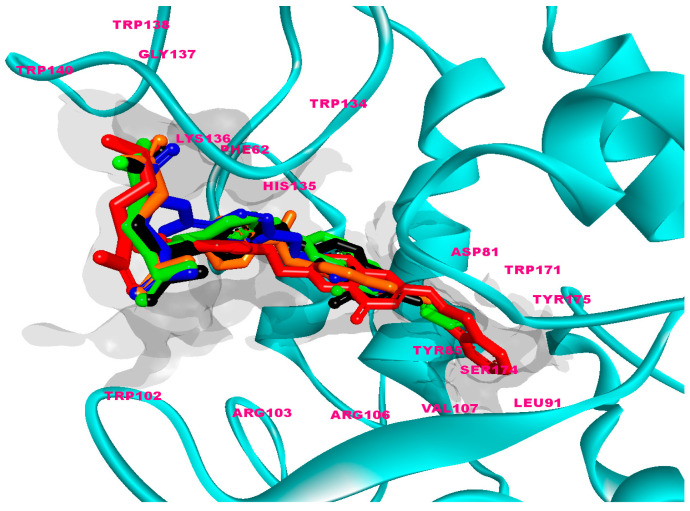
Superposition of FA (black), MTX (orange), PTX (blue), FOL03 (green), and FOL08 (red).

In this study, the structures of the FA analogs were designed by substituting the primary amine in the pteridine moiety with different functional groups: pyrrole (FOL01) [46], imidazole (FOL02) [47], tetrazole (FOL03) [48], piperidine (FOL04) [49], pyridine (FOL05) [50], pyrimidine (FOL06) [51], pyridazine (FOL07) [52], benzothiophene (FOL08) [53], indole (FOL09) [54], benzimidazole (FOL10) [55], purine (FOL11) [56], thiophene (FOL12) [57], thiazole (FOL13) [58], 1,3,4-thiadiazole (FOL14) [59], oxadiazole (FOL15) [60], oxetane (FOL16) [61], tetrahydropyran (FOL17) [62], oxazolidine (FOL18) [63], and furfuraldehyde ring (FOL19) [64]. These heterocyclic rings were chosen from the literature based on their role in anticancer activity. The chemical structures of FA, MTX, and PTX were acquired from the PubChem database (https://pubchem.ncbi.nlm.nih.gov/ (accessed on 22 February 2021)), whilst the 19 analogs were sketched using PerkinElmer ChemDraw 17.1 (Figure 1). Table 1 shows the free binding energy of FA, MTX, PTX, and the designed ligands. From the 19 FA analogs evaluated, FOL03 and FOL08 showed significantly lower binding energies than FA, although they bind at the same binding pockets as FA, MTX, and PTX (Figure 4).

Polar amino acids such as ASP81, LYS136, ARG103, HIS135, and non-polar amino acids such as TRP138, TRP140, and GLY137 played significant roles in stabilizing the FA–FRα complex, via H-bonding with the pteridine and the glutamic acid moieties (Figure S3). ASP81 is a dicarboxylic mono-amino acid and it is located at ~10 Å in the depth of the FRα pocket, as described earlier in Figure 3a. It forms two strong intermolecular H-bonds with FA; one with a pteridine ring at N4 at a distance of 1.67 Å and the other with the primary amino group N6 at a distance of 2.14 Å (Figure S3). This observation is consistent with previous studies that showed ASP81 interacted with the pteridine ring and is considered as a key contributor to high folate affinity [22,65]. In addition, two H-bonds were also formed between the carbonyl group at the C10 position in FA with HIS135 (1.92 Å) and ARG103 (2.21 Å). HIS135, which is located 5 Å from the surface to the middle of the FRα pocket was also observed to form paired H-bonds with the glutamic acid moiety of FA at N7 (1.83 Å) and O6 (1.87 Å). The second carboxylic acid group from the glutamic acid moiety formed H-bonds with the non-polar amino acids TRP138 (2.50 Å) and TRP140 (2.05 Å).

A non-covalent salt bridge interaction (ionic and H-bonds) was found to form between the cationic ammonium in LYS136 with the carboxylate ion (O5 and O6) of the glutamic acid moiety with a bond length of 2.53 Å and 2.02 Å, respectively. This type of interaction commonly occurs between LYS and GLU in a protein and considered the most energetic non-covalent interaction that can be formed between any two functional groups [66]. An analysis of the 2D and 3D diagrams also showed the formation of a strong intermolecular H-bond (1.93 Å) between the non-polar GLY137 with O4 from the glutamic acid moiety. This analysis confirmed the participation of FA in multiple intermolecular H-bonds with FRα. The interactions can be seen with ASP81 in the depth of the pocket, ARG103 and HIS135 in the middle, and LYS136, TRP138, TRP140, and GLY137 at the entrance of the binding pocket. These H-bonds, coupled with the hydrophobic interactions, contributed to the high stability of the binding. This result is indeed in agreement with Chen et al. who reported FA recognition by FRα [22].

Interestingly, MTX has a lower affinity toward FRα as compared to FA. The substitution of the carbonyl by an amino in the pteridine moiety reduces the binding affinity. Figure S4 illustrated the important bonds between MTX and FRα and their respective lengths. The fundamental difference between MTX and FA lies in the bending of the MTX scaffold in the FRα pocket. The bending of MTX causes it to lose two hydrogen bonds with ARG103 and HIS135, leading to a lower binding affinity as compared to FA.

The binding of PTX, on the other hand, is similar to FA and this was also observed in their similar binding energy. The substitution of the pyrazine ring by a pyrrole in PTX did not change the binding characteristics, as PTX was also shown to generate H-bonds with ASP81, THR82, and HIS135 from the pteridine and LYS136, GLY137, and TRP138 from the glutamic acid moiety (Figure S5).

**Figure 5:**
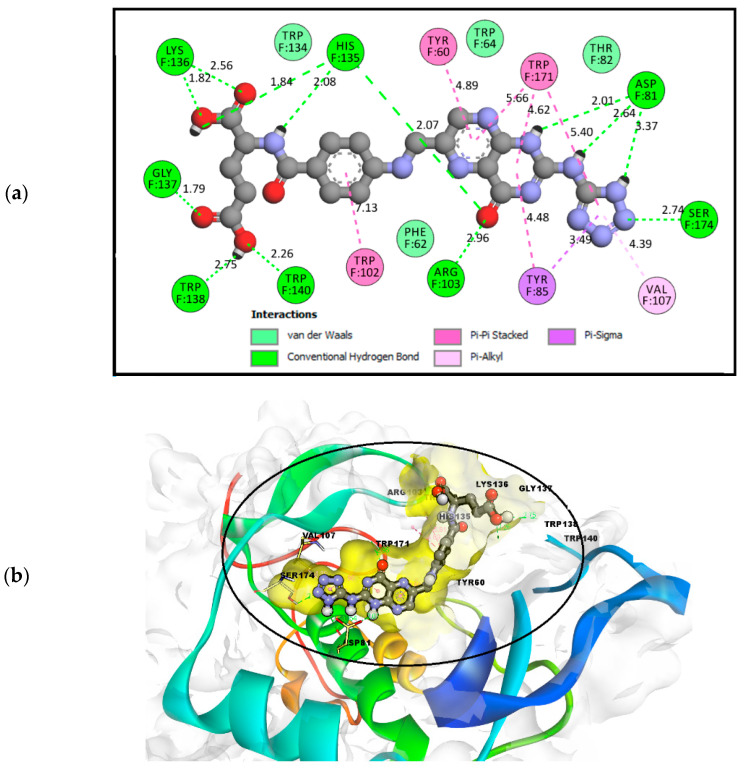
(**a**) Analysis of FOL03 (gray C, red O, and blue N) docked with FRα (PDB ID: 4LRH) presented as solid surface rendering. (**b**) The 2D binding site interaction between FOL03 and FRα.

FOL03 has the lowest binding energy, followed by FOL08 (Table 1). It forms hydrophobic interactions with the non-polar amino acids VAL107 and TRP171, and H-bonds with the polar amino acids ASP81, ARG103, and SER174 (Figure 5). The tetrazole ring that was conjugated to the primary amine of pteridine in FOL03 has a small geometry with aromatic properties and four electronegative nitrogen atoms as compared to other heterocyclic rings. This led to the increased electrostatic energy and improved affinity towards the receptor’s active site [67].

**Figure 6:**
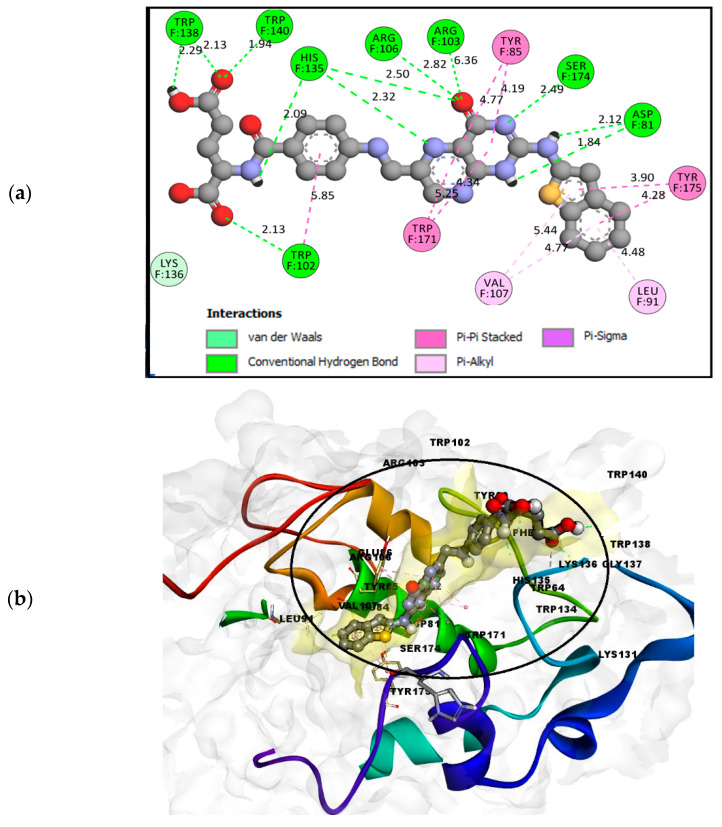
(**a**) Analysis model of the FOL08 (Gray C, red O, yellow S, and blue N) docked with FRα (PDB ID: 4LRH) presented as solid surface rendering. (**b**) The 2D binding-site interaction between FOL08 and FRα.

The interaction of FOL08 with FRα was comparable to FOL03, where it also demonstrated the ability to form H-bonds with ASP81 and SER174 (Figure 6). Surprisingly, new hydrophobic interactions were observed at the depth of the pocket with LEU91, VAL107, and TYR175. These amino acids were predicted to exist by the machine learning binding site determination server (P2Rank) in the receptor active site, as described earlier (Figure 2). In addition, the added benzothiophene ring also interacted with the receptor through van der Waals interactions, which increased the binding affinity.

### 2.3. Molecular Dynamics (MD) Simulation

#### 2.3.1. Root Mean Square Deviation (RMSD) Analysis

**Figure 7:**
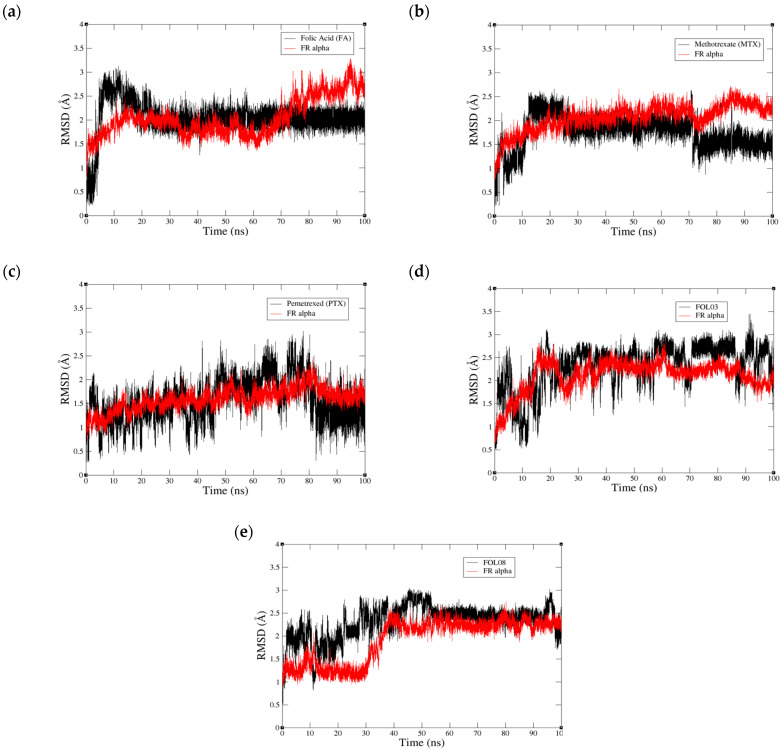
The root mean square deviation (RMSD) plots for the selected systems. (**a**) FRα–folic acid (FA), (**b**) FRα–methotrexate (MTX), (**c**) FRα–pemetrexed (PTX), (**d**) FRα–FOL03, and (**e**) FRα–FOL08. The ligands are in black and the FRα protein are in red.

**Figure 8:**
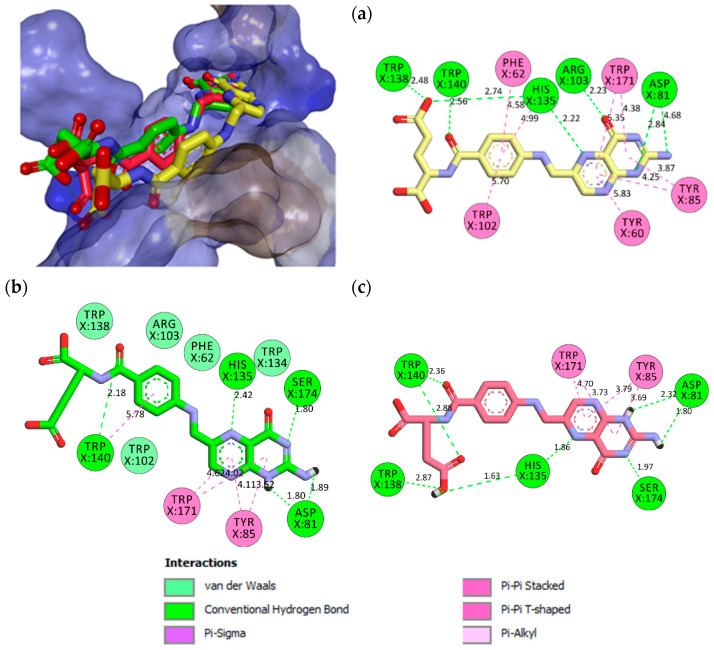
The FA ligand orientation and its interactions with FRα during the molecular dynamics (MD) simulation at 20 ns (**a**), 77.28 ns (**b**), and 94.96 ns (**c**).

As both FOL03 and FOL08 showed more negative binding energy (<−2 kcal/mol) than FA and PTX towards FRα, they were further subjected for 100 ns MD simulations. In addition, FRα systems containing FA, MTX, and PTX were also simulated for comparison. The stable complexes of FOL03–FRα and FOL08–FRα were compared to FA–FRα, MTX–FRα, and PTX–FRα complexes throughout the 100 ns MD simulations. In order to monitor the stability of the systems, the all-atom RMSD values of the five simulated systems (FOL03–, FOL08–, PTX–, MTX–, and FA–FRα complexes) during the 100 ns MD simulations were plotted (Figure 7). As shown in Figure 7a, the RMSD of FA (black plot) reached an equilibrium with a stable RMSD value of ~2.2 Å after 20 ns with similar fluctuations throughout the 100 ns. This observation is similar to that observed by Della-Longa et al. [68]. In the FA–FRα system, we can see that the average RMSD of the FRα varies between 1.5 Å and 3.3 Å over the simulation time. The increase in the RMSD value of the protein backbone is clearly observed after 60 ns, i.e., from 1.5 Å to 2.4 Å and continues to fluctuate between 2.4 Å and 3.0 Å after 77.28 ns until 100 ns of simulation. There are multiple FA orientations within the FRα pocket and examples are given for 20 ns, 77.28 ns, and 94.96 ns, as shown in Figure 8.

**Figure 9:**
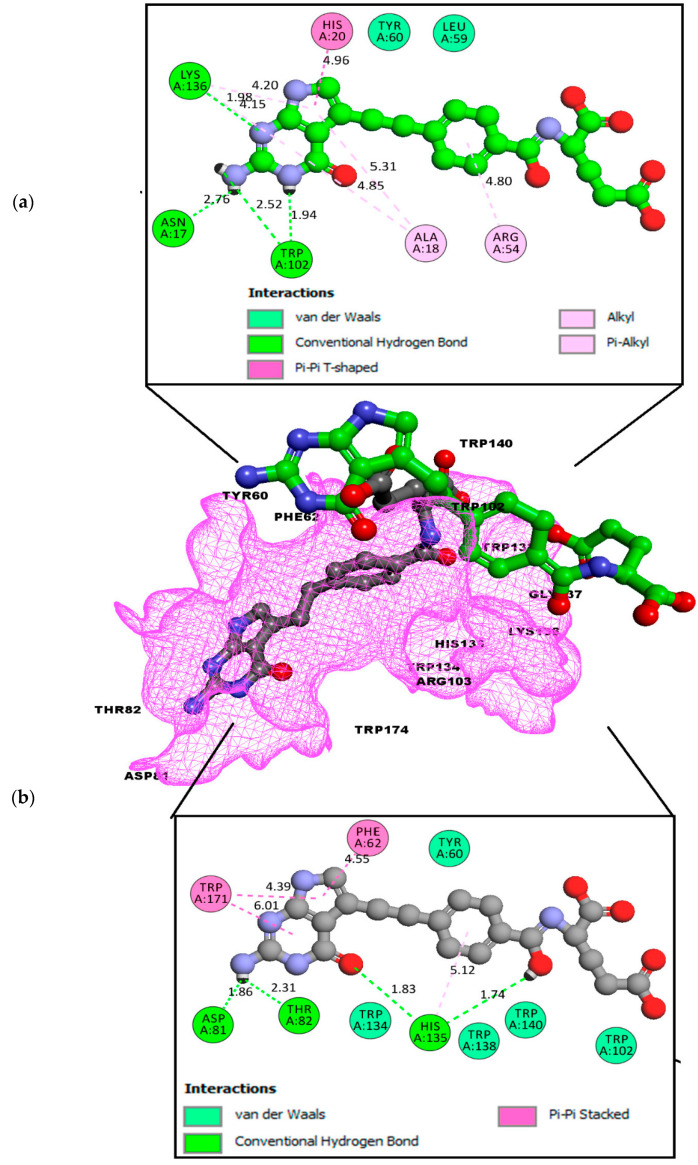
The FRα as extracted from the selected frames at (**a**) 82 ns and (**b**) 30 ns, from the 100 ns MD simulations. These models demonstrate how PTX left the binding pocket of FRα (pink) after losing the interaction with ASP81 at 82 ns.

In contrast, the RMSD value of MTX–FRα showed that MTX followed two distinct phases, but the difference did not significantly affect the stability of the complex. The first phase can be seen from 25 to 70 ns with an RMSD value of 2.0 Å, and the second phase from 70 to 100 ns with an average RMSD of 0.15 Å (Figure 7b). It is worth noting that the protein binding to MTX in the second phase showed a lower RMSD value than FA, which indicates that the protein is more stable after 70 ns. Similarly, in PTX, the RMSD profiles (Figure 7c) also demonstrated two distinct phases where the RMSD value gradually increased with a stable curve (~2.0 Å) until it reached 81 ns. Then, in the second phase, the average RMSD of PTX decreased to 1.0 Å, with big fluctuations in the RMSD plot, indicating that a highly unstable condition occurred within the pocket. However, it is important to note that the PTX lost its interaction with ASP81 inside the pocket as it left the pocket after ~82 ns of simulation and remained bound at the pocket entrance until the end of the simulation, as shown in Figure 9.

**Figure 10:**
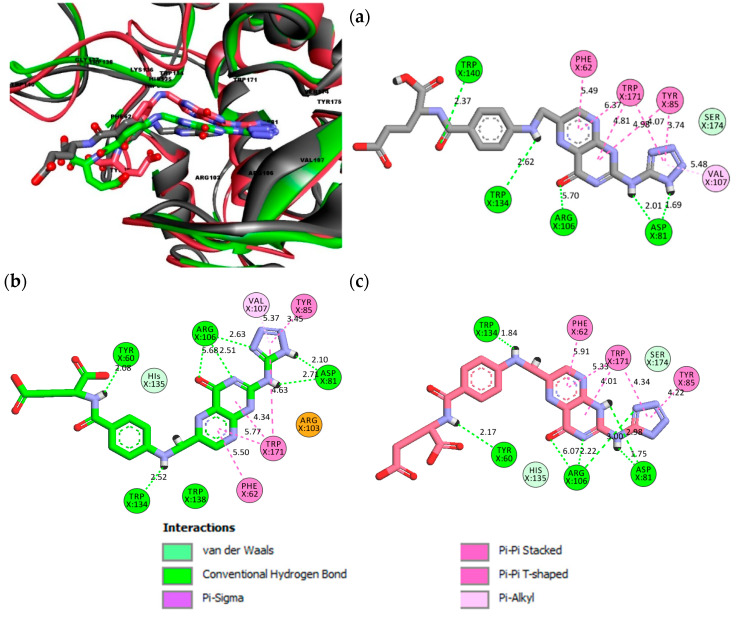
FOL03 orientation and its interactions with FRα during MD simulations at 68.24 ns (**a**), 86.20 ns (**b**), and 91.57 ns (**c**). This model demonstrates the behavior of the FOL03 interaction with ASP81 and the location of the amino acids in the active site of the FRα.

In the FOL03–FRα system (Figure 7d), equilibrium was reached after 20 ns with stable RMSD values of 2.0–3.0 Å. It is also noted that the RMSD value of FRα in the system (2.0–2.5 Å) is similar to that of FRα in the MTX–FRα system, indicating higher stability in FRα with FOL03 than FA after 70 ns of simulations. This is evidenced by a lack of changes in the FOL03 orientation relative to the protein at 68.24 ns, 86.20 ns, and 91.57 ns simulations, as presented in Figure 10.

**Figure 11:**
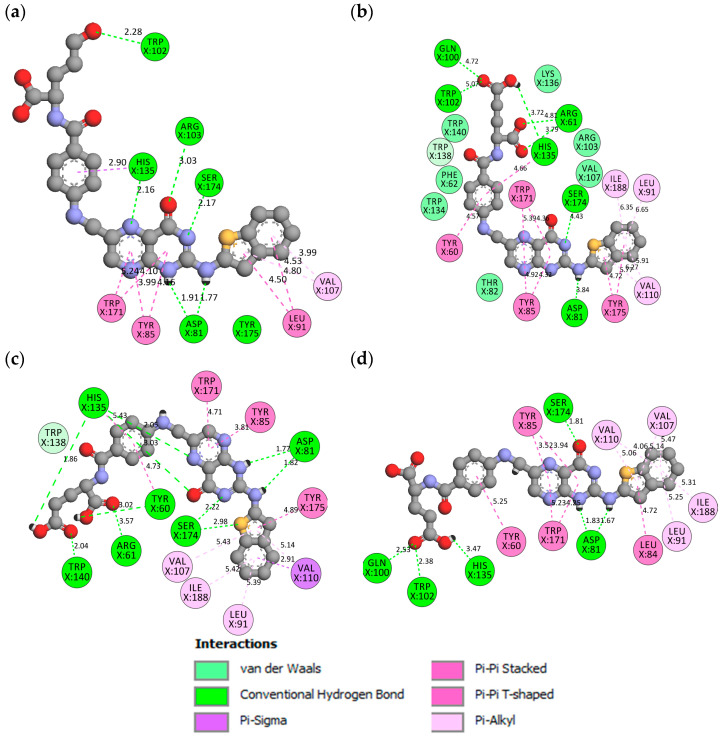
FOL08 orientations and its interactions with FRα during MD simulations at 30 ns (**a**), 45 ns (**b**), and 65 ns (**c**), and 95 ns (**d**). This model demonstrates the behavior of the FOL08 interaction with ASP81 and the location of the amino acids in the pocket of FRα.

The RMSD graph of FOL08–FRα (Figure 7e) showed that the system required 50 ns to achieve stability which is more than the other systems. Then, it reached a stable RMSD at an average of 2.3 Å until the end of the MD simulation time (100 ns). Figure 11 shows that the FOL08 scaffolds from the different time frames significantly overlapped, as it forms many interactions with the binding pocket throughout the simulation, thus implying its high stability, like FOL03. In general, all systems except PTX–FRα reached equilibrium with stable RMSD values ranging from 1.5 to 2.8 Å, suggesting that attaching heterocyclic rings (tetrazole and benzothiophene) to the pteridine ring of FA did not impair the stability of the complexes.

#### 2.3.2. Root Mean Square Fluctuation (RMSF) Analysis

**Figure 12:**
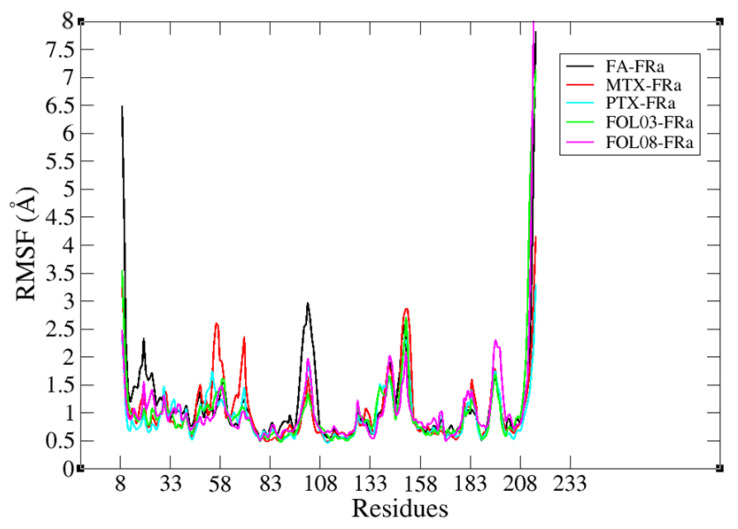
Root mean square fluctuation (RMSF) diagram results for complexes FA (black), MTX (red), PTX (cyan), FOL03 (green), and FOL08 (magenta).

**Figure 13:**
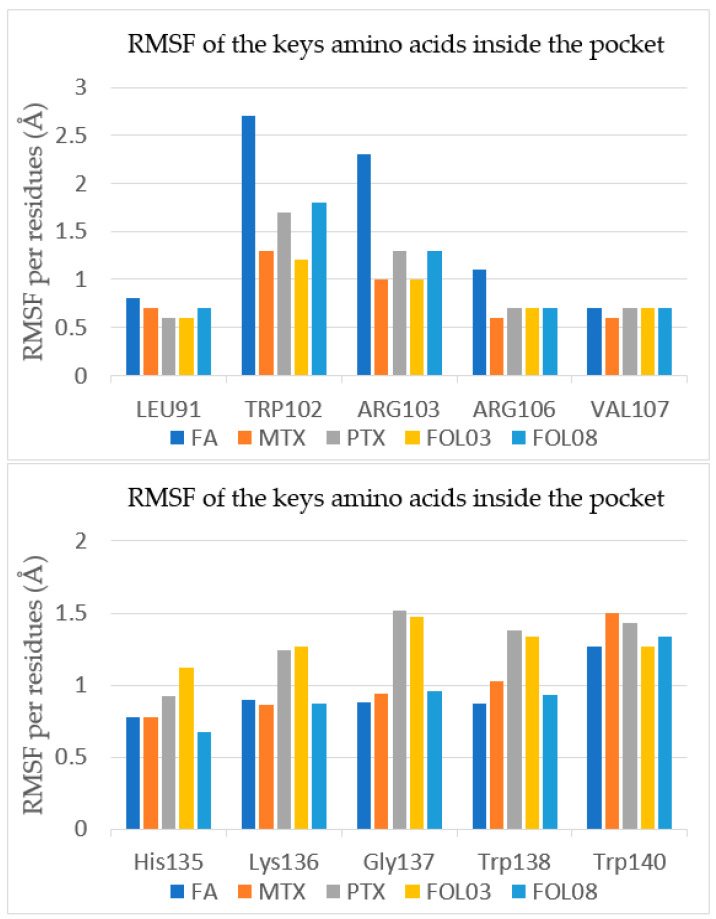
The RMSF values for the significant amino acids of the FRα active site after interacting with the selected ligands throughout a 100 ns MD simulation.

All fluctuations of the protein residues were very slight, less than 3.0 Å (Figure 12). The slight fluctuations indicate the formation of stable interactions between FA, MTX, PTX, FOL03, and FOL08 with FRα. The RMSF profiles of FOL03 and FOL08 complexes were similar to the FA–FRα complex. The fluctuations of residues near the docking pocket of FRα (such as ASP81, HIS135, GLY137, LYS136, ARG103, TYR60, TYR85, SER174, TRP 102, TRP138, TRP140, and TRP171) are very subtle, indicating that the binding of these analogs at the binding pocket is quite stable. However, there was a slight fluctuation of FA–FRα from residues 83–150 (region of fluctuations). It is interesting to note there are ten amino acids from the FRα active site in this region (LEU91, TRP102, ARG103, ARG106, VAL107, HIS13, LYS136, GLY137, TRP138, and TRP140), which helped to understand how the ligands (FA, MTX, PTX, FOL03, and FOL08) interacting inside the active site affected the RMSF values and stability of these residues (Figure 13). It is noted that TRP102, ARG103, and ARG106, which are the key amino acids, are mostly unaffected (more stable and lower RMSF values) by the binding of MTX, PTX, FOL03, and FOL08 compared to FA. This could be due to the different orientation adopted by the FA pteridine into the pocket compared to that of the analogs, which was reflected in the interactions of p-amino benzoic acid (PABA) and glutamate moieties with TRP102, ARG103, and ARG106.

#### 2.3.3. Binding Free Energy Calculation by Molecular Mechanics–Poisson-Boltzmann Surface Accessible (MM-PBSA)

Table 2 shows that the binding free energies of FRα–FA, FRα–MTX, FRα–PTX, FRα–FOL03, and FRα–FOL08 are favorable (−59.594, −45.120, −30.111, −73.620, and −79.677 kcal/mol, respectively). Both of the new FA analogs (FOL03 and FOL08) formed stronger interactions with FRα, with the binding free energy being more negative than that of FA, MTX, and PTX, and with the electrostatic interaction being a major contributor. From the perspective of the newly designed analogs, the result suggested that the most important part of the interaction with the FRα pocket is through creating strong electrostatic and hydrophobic interactions.

#### 2.3.4. Hydrogen Bond Properties

**Figure 14:**
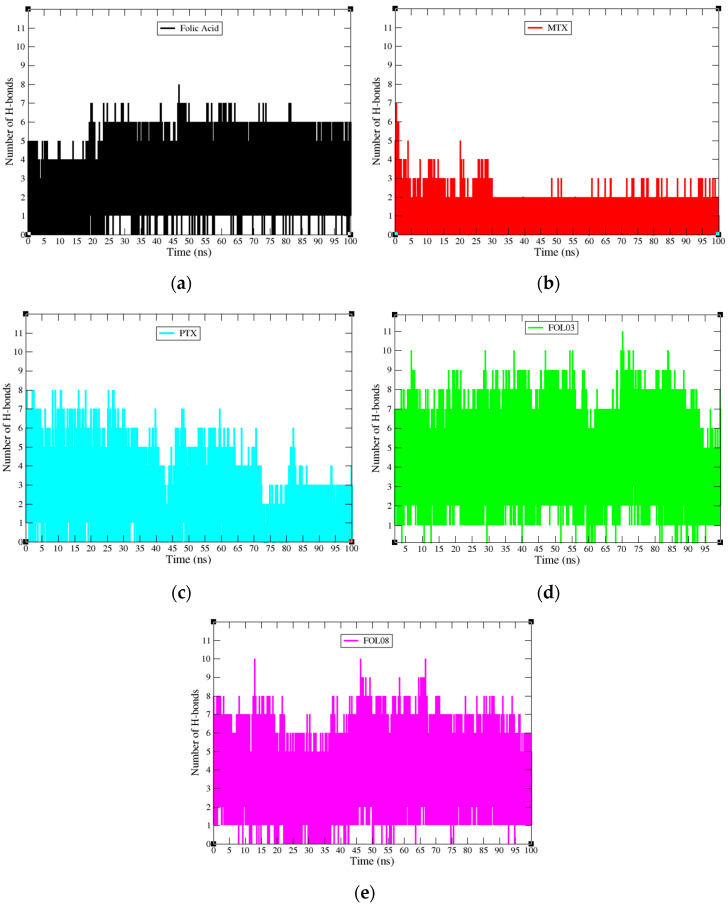
Hydrogen bond profiles during the 100 ns molecular dynamics simulations for complexes (**a**) FA (black), (**b**) MTX (red), (**c**) PTX (cyan) (**d**) FOL03 (green), and (**e**) FOL08 (magenta).

The average number of H-bonds and H-bond occupancy were analyzed for FA, MTX, PTX, FOL03, and FOL08 in the binding pocket of FRα throughout the MD simulation. ASP81 has been reported as the most important amino acid in the active site and has a key role in increasing the binding affinity, as well as the ability to hold the FA pteridine region deeply in the site [22,65]. Therefore, the analysis focused on the pteridine site for the selected FA analogs which can form interactions with ASP81 and the amino acids in the vicinity (Figure 14a–e). The hydrogen bond profile revealed that the initial stage of FRα–FA has five H-bonds and remained at four bonds until20 ns, during which the number of H-bonds increased to six bonds until 100 ns (Figure 14a). Correspondingly, at the starting time of FRα–MTX and FRα–PTX, the hydrogen bond profiles revealed seven H-bonds (FRα–MTX) and eight bonds (FRα–PTX). In the FRα–MTX system, the number of hydrogen bonds decreased from seven bonds at the first ns to two to five bonds until 30 ns, followed by a decrease to two to three bonds for the remaining time (Figure 14b). On the other hand, the hydrogen bond profile of FRα–PTX demonstrated good interactions (five to eight bonds) for 0–42 ns but, at 43 ns, it decreased to two H-bonds and then returned to a range of four to seven H-bonds until 72 ns. Then, the number of H-bonds decreased again to two to four H-bonds until 100 ns (Figure 14c). The H-bond profiles of the complexes (MTX and PTX) indicate that the systems are not capable of sustaining stable hydrogen bonding interactions in the binding pocket as in the FRα–FA system. In contrast, the hydrogen bond profiles of FRα–FOL03 and FRα–FOL08 displayed seven and eight H-bonds, respectively, at 0 ns and continued to range between five and ten H-bonds throughout the 100 ns simulation (Figure 14d,e). In addition, the bonding profiles showed stable average H-bonds ranging from six to eight H-bonds for the new FA analogs (FOL03 and FOL08) during the MD simulations, where both of them excelled over the FA interaction profile. The distinctive feature of the H-bond profile in FOL03 might have appeared because the FOL03 scaffold contains the tetrazole ring (four nitrogen atoms) which increased its electrostatic interaction with the amino acids in the depth of the pocket. Meanwhile, in FOL08, the scaffold includes a benzothiophene ring that forms a map of hydrophobic interactions with deep amino acids (LEU91, VAL107, and TYR175) which would have led to the pulling of FOL08 into the pocket and helped to generate interactions between polar amino acids such as SER174 and the FOL08 pteridine ring.

H-bonds contribute to the stability of the protein secondary structures and protein interaction with the ligands [65,69]. In Table 3, the H-bond occupancy, average distance, and angles were calculated for the selected systems to explore the consistent interactions between ligands (FA, MTX, PTX, FOL03, and FOL08) and ASP81 and the amino acids in the vicinity. In this study, the H-bonds were divided by their percentage of occupancy into strong (more than 60%), medium (between 30–60%), and weak hydrogen bonds (that occupied 10–30%) during the MD simulation [70]. The findings showed that there is a variation in the tendency of the H-bonds for selected ligands to bind with the FRα active site, and ASP81 is the key amino acid within it. Interestingly, during the MD simulation, FOL03 and FOL08 formed consistent hydrogen bonds compared to FA, while the H-bond occupancy of MTX and PTX was lower than FA. For FA–FRα, the results revealed a strong hydrogen bond between OD1 of ASP81 and the hydrogen atom (H2) at the N3 of the pteridine ring of FA with 61.28% occupancy during the 100 ns simulation, and with an average distance and angle of 2.83 Å and 159.70°, respectively. In addition, there is a moderate H-bond between OD2 of ASP81 and the hydrogen atom (H4) at the N5 of the primary amine of FA with 56.09% occupancy, and with an average distance and angle of 2.79 Å and 163.65°, respectively. The rest of the H-bond interactions, however, are relatively weak (Table 3). The findings also showed that both MTX and PTX were unable to maintain consistent H-bonds throughout the simulations. In contrast, both FOL03 and FOL08 form consistent hydrogen bonds with ASP81 of FRα; FOL03 with 75.40% and 74.93% occupancy to OD2 (ASP81) and 70.12% occupancy to OD1 (ASP81) and FOL08 with 63.39% and 41.74% occupancy to OD1 (ASP81) and 44.73% occupancy to OD2 (ASP81).

#### 2.3.5. Pocket Volume Calculations

**Figure 15:**
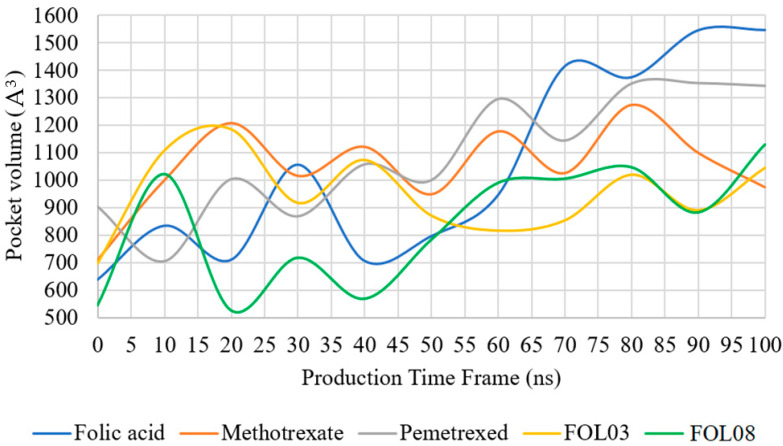
The volume of the FRα (PDB: 4LRH) binding pocket interacting with ligands (FA, MTX, PTX, FOL03, and FOL08) as a function of time during MD simulation.

Figure 15 shows the changes of pocket volume during MD simulations for all systems. Significant differences in the pocket size of FA are immediately evident at 65 ns. Although FA is highly stable inside the FRα, it tends to create a larger space inside the pocket. This could explain the sudden increase in the RMSD plot of FRα in the FA–FRα system at 65 ns (Figure 7a). Expansion in the binding pocket may also be an indication of the loss of ligand interactions where the ligand exited from the pocket [71], as observed in PTX after 80 ns of the MD simulation. It is also observed that after 65 ns, the volumes of binding sites for FOL03–FRα and FOL08–FRα were smaller than for the FA–FRα system. This may be due to strong electrostatic and hydrophobic, as well as H-bond, interactions within the pocket (Table 3 and Figure 14), which may have stabilized the ligands (FOL03 and FOL08) in the pocket during the MD simulation.

#### 2.3.6. General Effects of the Binding of FOL03 and FOL08 Inside FRα

From molecular docking, we have identified tetrazole- and benzothiophene-substituted analogs of FA, which have more negative binding energy (FOL03, −16.83 kcal/mol and FOL08, −16.24 kcal/mol, respectively) compared to FA (−13.20 kcal/mol), MTX (−11.87 kcal/mol), and PTX (−14.05 kcal/mol). These values are in agreement with the free binding energy calculated from MM-PBSA where FOL03 and FOL08 showed the most favorable binding energy (−73.62 and −79.68 kcal/mol, respectively) compared to FA, MTX, and PTX (−59.59, −45.12, and −30.11 kcal/mol, respectively).

The FA binding pocket in FRα is long and open, with the cavity shaped by TYR60, PHE62, ASP81, TYR85, TRP102, ARG103, ARG106, TRP134, HIS135, LYS136, GLY137, TRP138, TRP140, TRP171, SER174, and TYR175 [72]. In general, from the MD simulations, it was observed that there are no significant changes in the conformation of the FRα protein for all the systems throughout the 100 ns simulation, except in the FRα–FA system, where increased RMSD (from 1.5 to 3.0 Å) is seen after 65 ns. Pocket volume calculation confirmed that there is a doubling in the cavity’s original size in the active site (i.e., from 650 Å^3^ at the beginning of the simulation to about 1550 Å^3^ at the end of the simulation), which explains the loss of interactions seen in Figure 8. Interestingly, this increase did not significantly affect the stability of FA in the binding pocket. Although FA lost some interactions with important amino acid residues at the binding site, it is still able to maintain the H-bonds with ASP81, TRP138, TRP140, and HIS135. From the H-bond distances, it can also be seen that the H-bonds formed at the end of simulation are also generally stronger, thus compensating for the loss of other van der Waals interactions.

Both FOL03 and FOL08 also demonstrated more stable RMSD values (ranging from 1.5 to 2.8 Å) compared to FA, suggesting that attaching heterocyclic rings (tetrazole and benzothiophene) to the pteridine ring of FA might increase the stability of the complexes. The volumes of the active site for FOL03 and FOL08 were also smaller than for other systems, indicating stronger interactions with the protein active site residues compared to FA. FOL03 and FOL08 also formed more consistent hydrogen bonds compared to that of FA. Binding with these analogs also seems to stabilize the fluctuation of residues 91–107, where there are established interactions with residues LEU91, TRP102, ARG103, ARG106, and VAL107, which were not observed with FA, MTX, and PTX. As demonstrated by P2Rank, these amino acids create an additional cavity within the active site, thus allowing the heterocycle to fit and stabilize firmly within the active site.

### 2.4. ADMET Prediction

Table 4 displays the predicted pharmacokinetic profile (ADMET) of FOL03 and FOL08 compared to the controls (FA, MTX, and PTX). Scrutiny of the outcomes revealed that FOL03 and FOL08 possessed desirable ADME properties, where both are not able to penetrate the blood–brain barrier (BBB) and the central nervous system (CNS). In addition, it can be seen from Table 4 (metabolism) that FOL03 and FOL08 do not influence or inhibit the enzymes of cytochrome P450, so it can be expected that both analogs are unlikely to be metabolized in the body. The predicted toxicity of the analogs also showed that these compounds have a relatively lower acute toxicity risk compared to the controls.

## 3. Materials and Methods

### 3.1. Determination of the Size of the Binding Site

P2Rank and Depth web servers were used to assess the binding site coordinates and receptor residues (active site) for the 4LRH.PDB crystal complex with FA. For P2Rank service tool prediction, the crystal 4LRH.PDB was uploaded to the P2Rank web service and submitted to the pipeline server to start the prediction. For the Depth server prediction, the crystal 4LRH.PDB was uploaded similarly. Then, the number of solvent cycles was set to 25, the radius of the solvent neighborhood to 4.2 Å, and the minimum number of the neighborhood to 4 residues. For the purpose of obtaining the maximum total residue depth and eliminating the largest number of solvent molecules in the cavity, the probability threshold of the cavity was changed from 0.8 to 0.5, and the remaining parameters were kept as default. The process was eventually submitted to the server and the details were tracked and are presented in Figure 3.

### 3.2. Molecular Docking

The 3D structure of human FRα in complex with FA (PDB: 4LRH; 2.80 Å) [22] was retrieved from the Research Collaboratory for Structural Bioinformatics (RCSB) Protein Data Bank (http://www.rcsb.org/ (accessed on 22 February 2021)) [73]. The co-crystallized FA was taken out from the complex and saved as a PDB file using BIOVIA Discovery Studio Visualizer 16.1 and assigned with Gasteiger charges using AutoDockTools 1.5.6 and later redocked to the protein as a control docking using AutoDock 4.2. Other heteroatoms, including water molecules which are present in the crystal structure, were also eliminated. Furthermore, BIOVIA Discovery Studio Visualizer 16.1 was utilized to add all hydrogen atoms and protonate the amino acids that have ionizable side chains at physiological pH 7.00.

Polar hydrogens and Kollman charges were added to FRα and saved as PDBQT format using AutoDockTools 1.5.6. Meanwhile, the chemical structures of FA, MTX, PTX, and the nineteen FA analogs (Figure 1) were subjected to energy minimization using the Molecular Mechanics 2 (MM2) force field using PerkinElmer Chem3D 17.1. Then, FA, MTX, PTX, and the designed analogs were assigned with Gasteiger charges and saved in PDBQT format. The size of the grid box was set to 50 × 50 × 50, with the grid spacing set at 0.375 Å and centered on the binding pocket at coordinates 44.532, 41.058, 69.243 as *x*, *y*, *z*, respectively. Grid box parameters were then saved in grid parameter files (GPFs). For docking, AutoDock 4.2 was used, where the protein was set as rigid and ligand as flexible, the number of genetic algorithm runs was set to 150, population size 150, the maximum number of evals was 2,500,000 (medium), the maximum number of generations was 27,000, the Lamarckian genetic algorithm was chosen to perform this process, and the remaining parameters were kept as default and saved in the docking parameter files (DPFs).

Molecular interactions between the FA analogs and the active site of FRα were visualized using BIOVIA Discovery Studio Visualizer 16.1, which allows 2D and 3D visualization.

### 3.3. Molecular Dynamics (MD) Simulation

Molecular dynamics (MD) simulations using AMBER 18 [74] were performed for 100 ns for five FRα complexes. FOL03–FRα and FOL08–FRα systems were considered for this part according to their molecular docking results. On the other hand, FA, MTX, and PTX were employed to serve as controls where FA is the main control, MTX has less affinity than the control, and PTX has a higher affinity. The first steps involved the calculation of protein charge with the AMBER ff14SB force field, and describing the ligands using the general AMBER force field (GAFF) [74]. All ligands were subjected to AM1-BCC model charges using the ANTECHAMBER tools. TIP3P water was added in a cubic box with a volume of 10 × 10 × 10 Å [74]. The solvated protein–ligand systems were neutralized by adding Cl^−^ ions in order to counterbalance the charge of the resulting systems (Table 5).

Three minimization steps were conducted for 1000, 2000, and 5000 cycles of conjugate gradient [75] for the selected FA analogs, FA, MTX, and PTX complexes by using the AMBER18-SANDER module [76]. After the steps of minimization were performed, each of the FRα systems was heated from 0 to 310 Kelvin (K) in 3 heating steps prior to equilibration and production stages. Each heating step was carried out for 1 ns, starting from 0 K to 100 K in the first step, then in the second step from 100 K to 200 K, and finally from 200 K to 310 K, for all backbone atoms. During the heating process, NVT ensemble was used. Next, the equilibration of the macromolecule atoms and the surrounding solvent was performed for 2 ns in each step. Finally, the MD production step was carried out until 100 ns. Heating, equilibration, and production steps were run using PMEMD-AMBER 18 [75]. Both molecular docking and dynamics simulations were carried out using a computer with a 64-bit Ubuntu LTS 18.04 operating system, 64 GB of RAM with 24 cores Intel^®^ Xeon CPU E5-2620 2.40 GHz, and 2 cores Nvidia^®^ GeForce GTX Titan-X SSE2.

### 3.4. Free Binding Energy Calculation by MM-PBSA

All energetic analyses were done using a single trajectory approach, where the snapshots were taken for the protein–ligand complex, protein, and ligand of the performed MD trajectory. According to the MM-PBSA method [77], the Gibbs free binding energy ΔG_bind_ for every system can be conceptually defined as the following Equation (1):

(1) $$\Delta G_{\mathrm{bind}}=G_{\mathrm{RL}}-G_{R}-G_{L}$$

can be decomposed into contributions of different interactions and expressed as

(2) $$\Delta G_{\mathrm{bind}}=\Delta H-T\Delta S=\Delta E_{\mathrm{MM}}+\Delta G_{\mathrm{sol}}-T\Delta S$$

where

(3) $$\Delta E_{\mathrm{MM}}=\Delta E_{\mathrm{int}}+\Delta E_{\mathrm{ele}}\;\Delta E_{\mathrm{vdW}}$$

(4) $$\Delta G_{\mathrm{sol}}=\Delta G_{\mathrm{PB}}-\Delta G_{\mathrm{SA}}.$$

(5) $$\Delta G_{\mathrm{SA}}=\gamma \cdot SASA+b$$

where *G*_RL_, *G*_R_, and *G*_L_ are the free energy for the receptor-ligand complex, receptor, and ligand, respectively. Each term is calculated by averaging the energy of molecular mechanics (∆*E*_MM_), the solvation free energy (∆*G*_solv_), and the vibrational entropy term (*T*∆*S*) as in (2). ∆*E*_MM_ (3) denotes the average molecular mechanics energy contributed by bonded (*E*_int_) and nonbonded (*E*_vdw_ and *E*_EEL_) terms. ∆*G*_solv_ (4) is the solvation free energy given by ∆*G*_PB_, polar solvation free energy evaluated using the Poisson-Boltzmann equation, and ∆*G*_SA_, nonpolar contribution to solvation free energy from the surface area. ∆*G*_SA_, in turn, is estimated by the solvent accessible surface area (SASA).

The free binding energy difference for the FA analogs and folate receptor was measured with the help of molecular mechanics/Poisson–Boltzmann surface area (MM/PBSA) with negligible contribution of entropy energy for the systems [78]. In this study, the MMPBSA.py Python module as part of the AMBER18 bundle was used to calculate the binding energies’ differences for the selected systems. The energy was calculated for all MD trajectory times (100 ns), with 1000 frames extracted with an interval of 100 ps, salt concentration of 0.150 M, and with no quasi-harmonic entropy approximation.

### 3.5. Pocket Volume (POVME) Algorithm

Eleven frames were extracted from the production trajectory files every 10 ns using UCSF Chimera 1.13 [79]. Protein chains from all frames were superimposed using BIOVIA Discovery Studio 16.1 with default parameters and saved as a single PDB file [80]. Binding pocket volume calculations were computed using the POVME 2.2 Python script with a sphere of points 13.0 Å in radius, with the coordinates of FA (41.521627, 27.455255, 44.589373), MTX (32.534836, 33.296836, 46.676309), PTX (41.926611, 33.246389, 45.942815), FOL03 (39.164054, 38.965304, 43.397518), and FOL08 (37.169094, 31.399891, 45.128953) as *x*, *y*, and *z*, respectively. Grid spacing was set to 1.0 Å, while the distance cutoff was 1.09 Å [81].

### 3.6. ADMET Prediction

The prediction of the ADMET toxicity properties was performed using web service tools, pkCSM (http://biosig.unimelb.edu.au/pkcsm/ (accessed on 22 February 2021)) [82], which allows for predicting the mutagenicity (Ames test), carcinogenicity, the BBB permeability, and many other characteristics for the potential ligands against the controls (FA, MTX, and PTX), while the human intestinal absorption was examined using PreADMET (https://preadmet.bmdrc.kr/ (accessed on 22 February 2021)) [83]. The two-dimensional chemical structures of FA, MTX, PTX, FOL03, and FOL08 were converted to SMILES format and submitted to the web tools for their property prediction.

## 4. Conclusions

Nineteen FA analogs with various heterocyclic rings were designed and docked against FRα. Eleven out of 19 FA analogs have shown stronger binding energies than that of FA and two out of the eleven analogs had better binding affinities than PTX (more than −2 kcal/mol). FOL03, which has a tetrazole ring substitute, has the most negative binding energy to FRα (−16.83 kcal/mol), followed by the benzothiophene-substituted analog, FOL08 (−16.26 kcal/mol). The results also revealed new interactions of the analogs with SER174, TYR175, LEU91, and VAL107 located in the inner region of the FRα active site. However, such interactions were not seen with FA, MTX, and PTX. These observations indicate the importance of heterocyclic rings in enhancing the binding affinity of new FA analogs toward FRα. The interactions of FOL03 and FOL08 with FA, MTX, and PTX in the FRα binding pocket were further compared using MD simulation for 100 ns. The conformational analysis and orientations of the complexes showed a clear convergence, where both ligands entered the pocket from the pteridine region, and the glutamic region was positioned at the opening of the folate receptor pocket. Additionally, FOL03 and FOL08 systems, reaching their equilibrium state with stable RMSD values, have further confirmed the hypothesis of the potential of heterocyclic rings like tetrazole and benzothiophene by not impairing the stability of the systems. Intriguingly, MM-PBSA binding free energy calculations, H-bond analyses, and pocket volume calculations showed that FOL03 and FOL08 formed stronger binding interactions and had more negative free binding and electrostatic energies compared to FA and PTX, due to the key role of the heterocyclic rings which enhanced the electrostatic attractions between the FA analogs and the amino acid at the depth of the pocket. ADMETox predictions have also showed that the two designed ligands (FOL03 and FOL08) have desirable properties which indicate that they might be safe and have good pharmacokinetic and pharmacodynamic profiles. However, due to the time frame and resources available, the effort made here presented a theoretical prediction of features required for potential lead candidates. Many aspects to reach the clinical stage should be investigated and more work should be done so that current efforts are not left unfinished. It is suggested that the compounds can be synthesized using bio-isosteric replacement and analog design. Having the compounds synthesized will allow for the confirmation of activity to be ascertained. These two compounds should not only be considered for lead optimization but also could be used in the conjugation of nanoparticles for drug delivery for enhanced FA recognition by FRα, especially in the treatment of cancer.

## Figures and Tables

**Table 1:** Free binding energy (FBE), inhibition constant (K_i_) of FA, MTX, PTX, and the 19 FA analogs.

Compound	FBE (kcal/mol)	K_i_ (Picomolar pM)
**FA**	−13.20	209.24
**MTX**	−11.87	2000
**PTX**	−14.05	37.88
**FOL01**	−15.71	3.060
**FOL02**	−15.79	2.67
**FOL03**	−16.83	0.460
**FOL04**	−15.84	2.440
**FOL05**	−15.86	2.39
**FOL06**	−15.88	2.28
**FOL07**	−15.64	3.41
**FOL08**	−16.24	1.48
**FOL09**	−14.34	30.88
**FOL10**	−15.56	3.92
**FOL11**	−14.40	27.68
**FOL12**	−13.84	71.24
**FOL13**	−14.75	15.40
**FOL14**	−14.87	12.49
**FOL15**	−15.71	3.06
**FOL16**	−14.82	13.73
**FOL17**	−15.24	6.74
**FOL18**	−14.81	13.84
**FOL19**	−14.98	10.43

**Table 2:** Binding free energies from Molecular Mechanics–Poisson-Boltzmann Surface Accessible MM-PBSA for FA, MTX, PTX, FOL03, and FOL08 with FRα from MD simulation trajectories. Molecular docking values from AutoDock for the complexes are also included in the table.

Complex with FRα	$\Delta G_{bind^{*}}$ kcal/mol	VDWLS kcal/mol	EEL kcal/mol	G_polar_ kcal/mol	G_non-polar_ kcal/mol	AutoDock kcal/mol
FA	−59.59 ± 0.17	−55.84 ± 0.15	−91.97 ± 0.28	94.35 ± 0.21	−6.12 ± 0.01	−13.20
MTX	−45.12 ± 0.18	−60.71 ± 0.12	−48.05 ± 0.39	69.98 ± 0.31	−6.34 ± 0.01	−11.87
PTX	−30.11 ± 0.36	−41.38 ± 0.24	−44.14 ± 0.48	60.57 ± 0.40	−5.16 ± 0.03	−14.05
FOL03	−73.62 ± 0.21	−61.47 ± 0.13	−134.73 ± 0.40	129.16 ± 0.28	−6.58 ± 0.01	−16.83
FOL08	−79.68 ± 0.21	−75.96 ± 0.15	−99.95 ± 0.38	104.81 ± 0.27	−8.57 ± 0.01	−16.24

$\Delta G_{bind^{*}}$: binding free energy, VDWLS: van der Waals, EEL: electrostatic, G_polar_: polar solvation energy, G_non-polar_: non-polar solvation energy.

**Table 3:** Hydrogen bond analysis for 100 ns of MD simulation for FA, MTX, PTX, FOL03, and FOL08 within the FRα active site.

Code	H-Bond Acceptor (atom@res)	H-Bond Donor (atom@H)	Donor (atom@res)	H-Bond Occupancy (%)	Average Distance (Å)	Average Angle (°)
FA	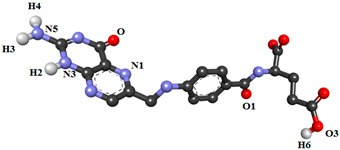					
	ASP81@OD1	FA@H2	FA@N3	61.28	2.83	159.70
	ASP81@OD2	FA@H4	FA@N5	56.09	2.79	163.65
	ASP81@OD2	FA@H3	FA@N5	16.67	2.79	163.66
	ASP81@OD1	FA@H3	FA@N5	13.01	2.81	161.64
	ASP81@OD2	FA@H2	FA@N3	11.26	2.85	152.98
	FA@O	ARG103@HH12	ARG103@NH1	17.18	2.84	149.17
	HIS135@O	FA@H6	FA@O3	56.89	2.72	156.37
	FA@N1	HIS135@HE2	HIS135@NE2	21.31	2.92	162.11
	FA@O1	TRP140@HE1	TRP140@NE1	43.77	2.83	148.12
MTX	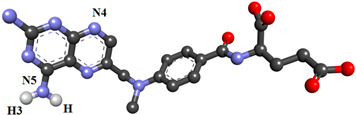					
	ASP81@OD1	MTX@H3	MTX@N5	22.89	2.82	152.41
	ASP81@OD1	MTX@H	MTX@N5	22.15	2.81	153.59
	ASP81@OD2	MTX@H	MTX@N5	13.03	2.83	153.19
	MTX@N4	ARG103@HH12	ARG103@NH1	10.22	2.91	147.23
	MTX@N4	HIS135@HE2	HIS135@NE2	10.96	2.91	158.30
PTX	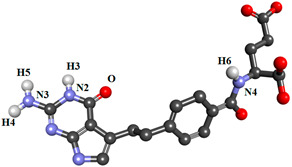					
	ASP81@OD1	PTX@H4	PTX@N3	11.46	2.81	159.08
	PTX@O	HIS135@HE2	HIS135@NE2	45.99	2.84	160.57
	HIS135@O	PTX@H6	PTX@N4	20.06	2.86	153.34
FOL03	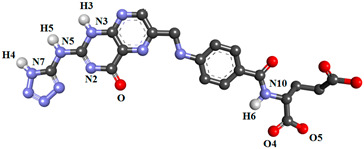					
	ASP81@OD2	FOL03@H5	FOL03@N5	75.40	2.82	157.41
	ASP81@OD2	FOL03@H3	FOL03@N3	74.93	2.76	151.43
	ASP81@OD1	FOL03@H4	FOL03@N7	70.12	2.76	148.54
	ASP81@OD1	FOL03@H5	FOL03@N5	28.07	2.86	147.80
	TYR60@O	FOL03@H6	FOL03@N10	23.44	2.89	157.28
	FOL03@O4	ARG61@HE	ARG61@NE	18.90	2.86	157.00
	FOL03@O5	ARG61@HH21	ARG61@NH2	16.70	2.88	157.43
	FOL03@O FOL03@O FOL03@N2	ARG107@HH11 ARG107@HH21 ARG107@HH21	ARG107@NH1 ARG107@NH2 ARG107@NH2	26.63 18.56 13.63	2.84 2.85 2.92	149.99 147.16 156.63
FOL08	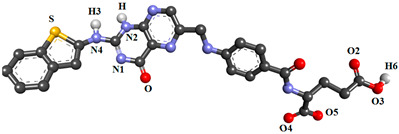					
	ASP81@OD1	FOL08@H3	FOL08@N4	63.39	2.79	163.29
	ASP81@OD2	FOL08@H	FOL08@N2	44.73	2.83	152.71
	ASP81@OD1	FOL08@H	FOL08@N2	41.74	2.83	150.17
	ASP81@OD2	FOL08@H3	FOL08@N4	32.20	2.77	162.82
	FOL08@O4	ARG61@HH21	ARG61@NH2	15.71	2.84	158.32
	HIS135@O	FOL08@H6	FOL08@O3	33.81	2.71	158.35
	FOL08@N	HIS135@HE2	HIS135@NE2	18.73	2.92	160.57
	FOL08@O2	TRP140@HE1	TRP140@NE1	16.08	2.86	156.43
	FOL08@N1	SER174@HG	SER174@OG	32.52	2.89	164.20
	FOL08@O	SER174@HG	SER174@OG	10.11	2.80	155.58

**Table 4:** Predicted ADMET properties of the ligands (FA, MTX, PTX, FOL03, and FOL08) using pkCSM and PreADMET.

Property	Model Name	Predicted Value				
		FA	MTX	PTX	FOL03	FOL08
**Absorption**	Water solubility (log mol/L)	−2.88	−2.859	−2.842	−2.892	−2.905
	Caco2 permeability (log cm/s)	−0.877	−0.77	−0.954	−0.92	−0.667
	Human intestinal absorption (% absorbed)	17.745	35.716	37.981	7.719	76.253
	Skin permeability (log Kp)	−2.735	−2.735	−2.735	−2.735	−2.735
	P-glycoprotein substrate	Yes	Yes	Yes	Yes	Yes
	P-glycoprotein I inhibitor	No	No	No	No	No
	P-glycoprotein II inhibitor	No	No	No	No	No
**Distribution**	Human volume of distribution (log L/kg)	0.046	−0.883	−0.927	−0.548	−0.720
	Human fraction unbound (Fu)	0.370	0.183	0.160	0.276	0.127
	Blood Brain Barrier (BBB)permeability (log BB)	−1.615	−1.865	−1.442	−3.458	−2.372
	CNS permeability (log PS)	−4.262	−3.818	−4.022	−7.265	−4.174
**Metabolism**	CYP2D6 substrate	No	No	No	No	No
	CYP3A4 substrate	No	No	No	No	No
	CYP1A2 inhibitor	No	No	No	No	No
	CYP2C19 inhibitor	No	No	No	No	No
	CYP2C9 inhibitor	No	No	No	No	No
	CYP2D6 inhibitor	No	No	No	No	No
	CYP3A4 inhibitor	No	No	No	No	No
**Excretion**	Total clearance (log ml/min/kg)	0.527	0.378	0.285	−0.196	−0.111
	Renal OCT2 substrate	No	No	No	No	No
**Toxicity**	Ames toxicity	No	No	No	No	No
	Max. human tolerated dose (log mg/kg/day)	−0.586	−0.827	−0.292	0.366	0.489
	hERG I inhibitor	No	No	No	No	No
	hERG II inhibitor	No	Yes	No	No	No
	Oral rat acute toxicity (LD50) (mol/kg)	2.670	2.713	2.585	2.483	2.501
	Oral rat chronic toxicity (LOAEL) (log mg/kg_bw/day)	3.153	3.112	3.111	4.876	3.152
	Skin sensitization	No	No	No	No	No
	*T. pyriformis* toxicity (log ug/L)	0.285	0.285	0.285	0.285	0.285
	Minnow toxicity (log mM)	4.009	2.384	2.867	4.886	1.221

**Table 5:** MD system setup details.

System	Total Numberof Atoms	Number of Heteroatoms	Water Atoms	Neutralizing Atoms
FRα + FA	29,808	3634	9033	3 Cl^−^
FRα + MTX	29,812	3639	9033	3 Cl^−^
FRα + PTX	29,808	3643	9033	3 Cl^−^
FRα + FOL03	29,807	3635	9031	3 Cl^−^
FRα + FOL08	29,809	3643	9029	3 Cl^−^

## Supplementary Materials

The following are available online, Figure S1. The 3D crystal structure of folate receptor alpha (4LRH.PDB) complex with folic acid (C atoms in green, N in blue, and O in red color) in the active binding site (Rose color), Figure S2. FA-FRα binding model. Inset is the superimposition of co-crystallized FA from 4LRH.PDB (Orange C, red O, and blue N) and docked FA (Green C, red O, and blue N) with RMSD = 0.90 Å, Figure S3. a. 3D visualization for FA-FRα (4LRH.PDB) interactions using hydrophobicity solvent model. Amino acid residues surrounding ASP81 that formed a small cavity in the depth was highlighted as the red circle (generated by BIOVIA Discovery Studio Visualizer 16.1). b. 2D image of amino acid residues involved in the FA- FRα interactions (Gray C, red O, and blue N). The symbol F in (**b**) refers to the amino acids from FRα, Figure S4. 2D image of the residues of amino acids involved in the interactions between FRα and MTX (Gray C, red O, and blue N). The symbol F in (**b**) refers to the amino acids from FRα, Figure S5. 2D image of the residues of amino acids involved in the interactions between FRα and PTX (Gray C, red O, and blue N). The symbol **F** in (**b**) refers to the amino acids from FRα.
