# Supplementary material for: Heterocyclic Substitutions Greatly Improve Affinity and Stability of Folic Acid towards FRα. an In Silico Insight
Source: Molecules. 2021 Feb 18;26(4):1079. doi: 10.3390/molecules26041079 (PMC7922218; doi:10.3390/molecules26041079)
Supplement: Supplementary file 1 [file molecules-26-01079-s001.pdf]

# Supplementary Materials

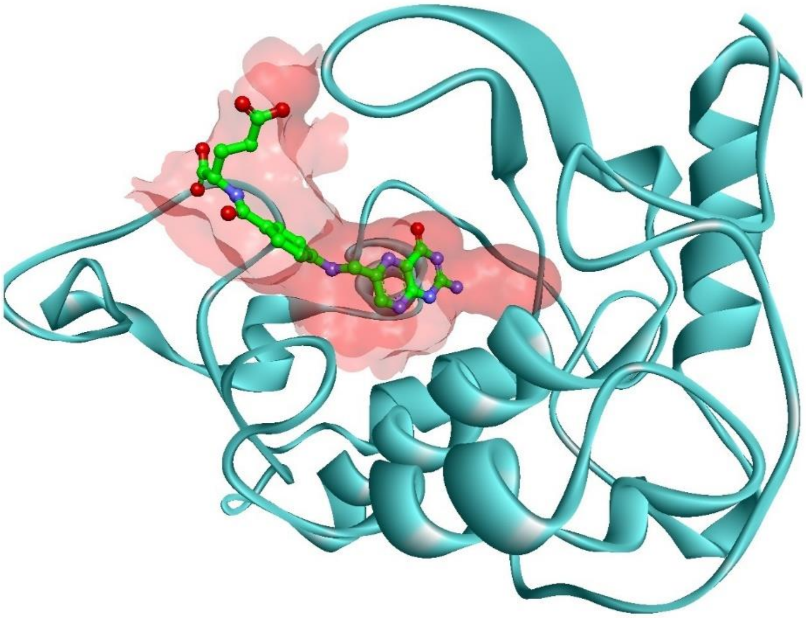

**Figure S1.** The 3D crystal structure of folate receptor alpha (4LRH.PDB) complex with folic acid (C atoms in green, N in blue, and O in red color) in the active binding site (Rose color).

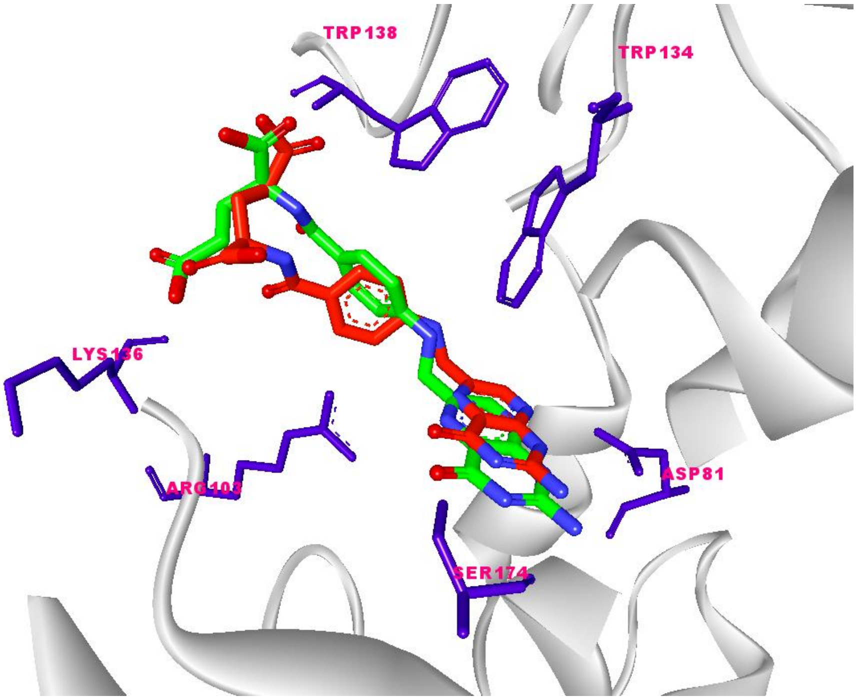


**Figure S2.** FA-FR$\alpha$ binding model. Inset is the superimposition of co-crystallized FA from 4LRH.PDB (Orange C, red O, and blue N) and docked FA (Green C, red O, and blue N) with RMSD = 0.90 Å.

a.

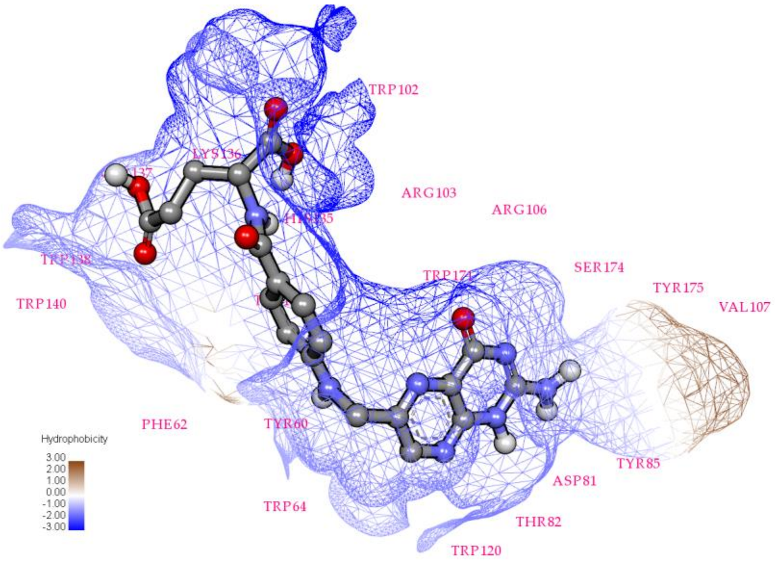


b.

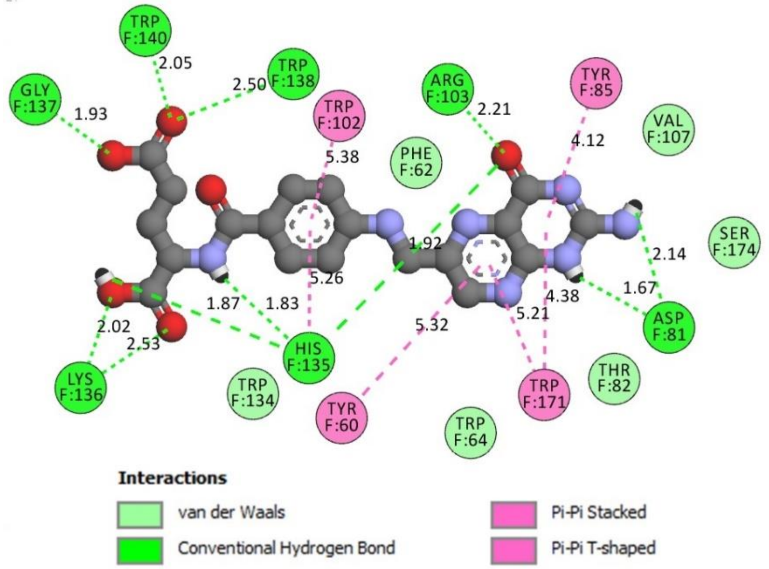


**Figure S3.** a. 3D visualization for FA-FR$\alpha$ (4LRH.PDB) interactions using hydrophobicity solvent model. Amino acid residues surrounding ASP81 that formed a small cavity in the depth was highlighted as the red circle (generated by BIOVIA Discovery Studio Visualizer 16.1). b. 2D image of amino acid residues involved in the FA-FR$\alpha$ interactions (Gray C, red O, and blue N). The symbol F in (b) refers to the amino acids from FR$\alpha$.

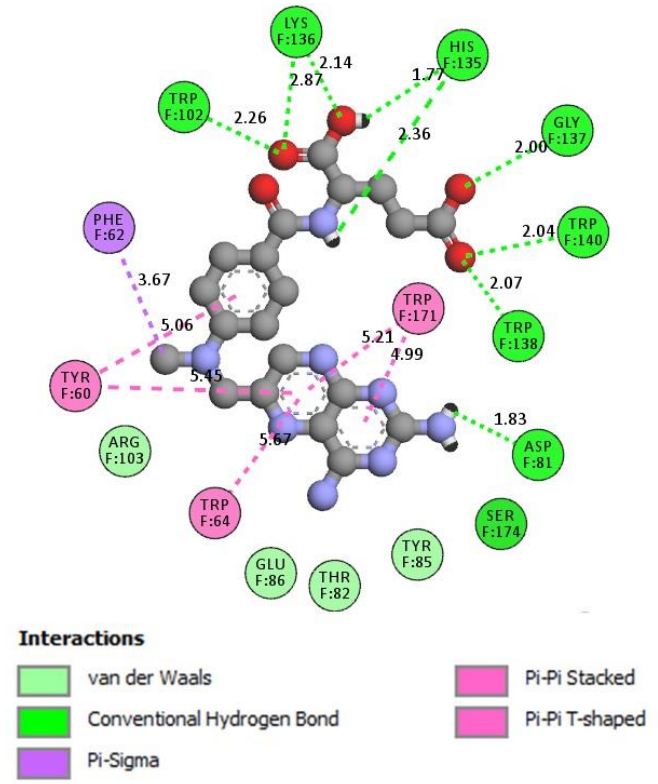


**Figure S4.** 2D image of the residues of amino acids involved in the interactions between FR$\alpha$ and MTX (Gray C, red O, and blue N). The symbol F in (b) refers to the amino acids from FR$\alpha$.

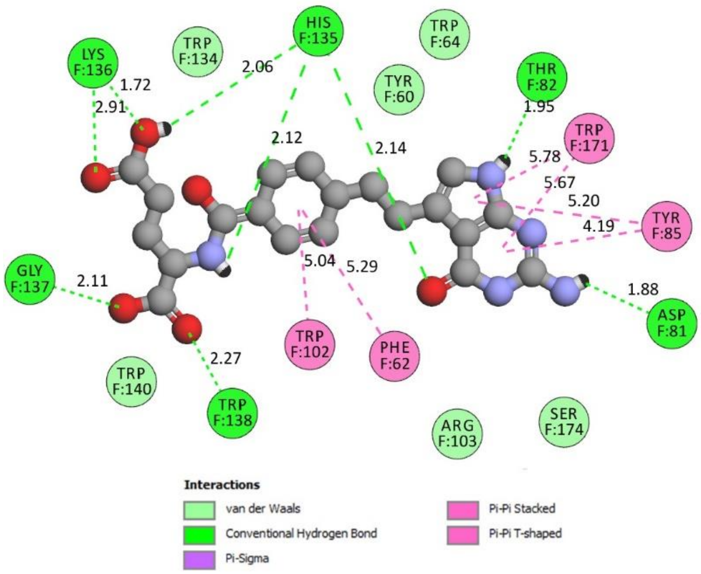


**Figure S5.** 2D image of the residues of amino acids involved in the interactions between FR$\alpha$ and PTX (Gray C, red O, and blue N). The symbol **F** in (b) refers to the amino acids from FR$\alpha$.
